# Short-term right heart reverse remodeling with vericiguat in HFrEF: reductions in pulmonary arterial pressure and chamber dimensions

**DOI:** 10.3389/fcvm.2026.1894753

**Published:** 2026-09-14

**Authors:** Yaoting Zhang, Chengpeng Zhang, Chao Tang, Hui Li, Jing Zhu

**Affiliations:** Department of Cardiology, The Second Affiliated Hospital of Soochow University, Suzhou, Jiangsu, China

**Keywords:** heart failure, heart failure with reduced ejection fraction, pulmonary hypertension, right ventricular function, right ventricular-pulmonary arterial coupling, soluble guanylate cyclase stimulator, vericiguat

## Abstract

**Background:**

Heart failure is the end-stage of various cardiovascular diseases. Although a variety of drugs have slowed down the progression of the disease, the prognosis of heart failure patients remains unsatisfactory. Vericiguat can improve left ventricular function, but there is relatively less research on its effect on right ventricular function. This study aimed to evaluate whether vericiguat can improve the right ventricular function of heart failure patients.

**Methods:**

Patients with left ventricular ejection fraction < 45% who were hospitalized for the first time in the Department of Cardiology, The Second Affiliated Hospital of Soochow University from January 1, 2023, to December 31, 2024 were included and divided into two groups. The baseline and 3-month follow-up clinical characteristics, laboratory data, routine transthoracic echocardiogram parameters, and medication use of patients were collected.

**Results:**

A total of 90 eligible patients were finally included, with 60 cases in the vericiguat group and 30 cases in the control group. In the vericiguat group, right ventricular function improved significantly after treatment. The tricuspid annular plane systolic excursion to pulmonary artery systolic pressure (TAPSE/PASP) ratio increased significantly [0.51 ± 0.22 vs. 0.60 ± 0.22, *p* = 0.001]. Right atrial longitudinal dimension index significantly decreased [30.07 ± 5.80 mm/m^2^ vs. 28.57 ± 5.43 mm/m^2^, *p* = 0.001], as did the Right atrial transverse dimension index [23.21 ± 5.13 mm/m^2^ vs. 21.86 ± 4.40 mm/m^2^, *p* = 0.012] and right ventricular basal diameter index [22.48 ± 3.96 mm/m^2^ vs. 21.07 ± 3.59 mm/m^2^, *p* < 0.001]. Pulmonary arterial systolic pressure decreased significantly [41.47 ± 16.25 mmHg vs. 36.20 ± 14.32 mmHg, *p* = 0.006]. In the control group, no significant changes were observed in right heart function parameters. Multiple linear regression analysis showed that the increase in ΔTAPSE/PASP was not significantly associated with changes in left ventricular function.

**Conclusions:**

Vericiguat treatment was associated with a reduction in pulmonary artery systolic pressure, improves right ventricle-pulmonary artery coupling, and promotes structural reverse remodeling of the right atrium and right ventricle in heart failure patients. These right heart benefits occur independently of changes in left ventricular structure and function. Vericiguat showed good short-term safety.

## 1 Introduction

Heart failure (HF) represents a major global public health challenge. Heart failure with reduced ejection fraction (HFrEF) accounts for approximately 35.2% to 47.8% of all HF cases (1, 2). Among Asian populations, patients with HFrEF commonly present with a high burden of comorbidities, and the 1-year mortality rate can reach 10.6%. In China, the prevalence of HFrEF among adults is approximately 1.1%, with an estimated 3 million new cases annually. The mean age of hospitalized patients with HF is 67.9 years, and the 1-year readmission rate is as high as 32.8%. Hypertension and coronary artery disease(CAD) are the leading causes of HFrEF, accounting for 47.1% and 47.8% of cases, respectively. The presence of comorbid conditions, such as atrial fibrillation and diabetes, and the high risk of rehospitalization are associated with increased mortality. In-hospital all-cause mortality ranges from 8.5% to 22.4% (2). Evidence suggests that HF patients with concomitant right ventricular dysfunction have worse outcomes (3).

In patients with HFrEF, elevated left ventricular filling pressure can be transmitted backward to the pulmonary circulation, leading to pulmonary venous hypertension and, in some patients, secondary pulmonary vascular remodeling. The resulting increase in right ventricular afterload may cause right ventricular–pulmonary arterial uncoupling, right ventricular dilatation, and eventually right-sided heart failure. Right ventricular(RV) dysfunction is directly associated with adverse outcomes in patients with HF. A recent large-scale international study (the G-CHF study), which included 17,321 patients with HF across 40 countries, indicated that RV dilation and dysfunction were stronger predictors of death and HF hospitalization than reduced left ventricular ejection fraction (LVEF) (4). Pulmonary hypertension due to left heart disease is the most common form of pulmonary hypertension(PH), accounting for 35% to 60% of all PH cases. The prevalence of PH is approximately 40% in patients with heart failure with preserved ejection fraction (HFpEF) and nearly 80% in those with HFrEF. The presence of PH is associated with increased mortality and higher rates of hospitalization in patients with HF (5).

Vericiguat is an oral soluble guanylate cyclase (sGC) stimulator that activates the sGC-cyclic guanosine monophosphate (cGMP) pathway, thereby enhancing nitric oxide-mediated vasodilation, exerting antifibrotic effects, and improving myocardial metabolism and endothelial function. Its unique mechanism of action is independent of traditional neurohormonal antagonists such as angiotensin receptor neprilysin inhibitors (ARNIs) and β-blockers, and may synergistically enhance left ventricular contractility while attenuating myocardial fibrosis (6). The VICTORIA trial demonstrated that the addition of vericiguat to standard quadruple therapy significantly reduced the composite risk of cardiovascular death or HF hospitalization by 10% in patients with HFrEF and LVEF <45%, with the greatest benefit observed in patients who had experienced a recent worsening HF event (7). However, dedicated studies evaluating the effects of vericiguat on RV function and PH remain limited. Most recent studies have focused primarily on improvements in left ventricular performance and overall clinical outcomes (8). The effects of vericiguat on RV remodeling and the progression of PH have not been clearly elucidated. Therefore, this study aimed to determine whether vericiguat improves RV function and reduces pulmonary arterial pressure in patients with HFrEF and LVEF < 45%.

## 2 Methods

### 2.1 Study design

This single-center, retrospective, observational study was conducted in the Department of Cardiology at the Second Affiliated Hospital of Soochow University. Patients hospitalized for the first time with heart failure (LVEF <45%) between January 1, 2023 and December 31, 2024 were included. Baseline clinical characteristics, laboratory tests, standard transthoracic echocardiographic (TTE) parameters, and medication regimens were collected. Patients were followed for a period of 3 months. The primary objective was to evaluate the effects of vericiguat in combination with guideline-directed medical therapy (GDMT) compared with GDMT alone, on left ventricular(LV) function, RV function, and pulmonary arterial pressure in patients with LVEF <45%. Safety outcomes related to vericiguat were also assessed. The study protocol was approved by the Ethics Committee of the Second Affiliated Hospital of Soochow University (approval No. JD-HG-2025-081). The requirement for written informed consent was waived by the Ethics Committee because of the retrospective study design and the use of de-identified clinical data. The study was conducted in accordance with the Declaration of Helsinki.

### 2.2 Study population

#### 2.2.1 Inclusion criteria

Patients with LVEF <45% on standard TTE who met the diagnostic criteria were included for HF according to the Chinese Guidelines for the Diagnosis and Treatment of Heart Failure (9). Patients were assigned to the vericiguat group (receiving GDMT plus vericiguat) or the control group (GDMT alone) based on medication exposure. Matching was performed according to the underlying etiology of HF, including ischemic cardiomyopathy, dilated cardiomyopathy, arrhythmia, and hypertension, in a 15:8:5:2 ratio.

#### 2.2.2 Exclusion criteria

Patients were excluded if they: (1) were younger than 18 years or older than 85 years; (2) had rheumatic heart disease, congenital heart disease, hypertrophic cardiomyopathy, or advanced atrioventricular block (Mobitz type II second-degree or higher); (3) had a history of heart transplantation; concomitant malignancy; idiopathic pulmonary arterial hypertension; severe pulmonary disease; hematologic disorders; severe infectious disease; advanced hepatic dysfunction (Child-Pugh class C); or end-stage renal disease (chronic kidney disease stage 5); or (4) had substantial missing data, such as absence of baseline or follow-up imaging (echocardiography) or key laboratory values. Figure 1 illustrates the detailed workflow of participant screening.

**Figure 1:**
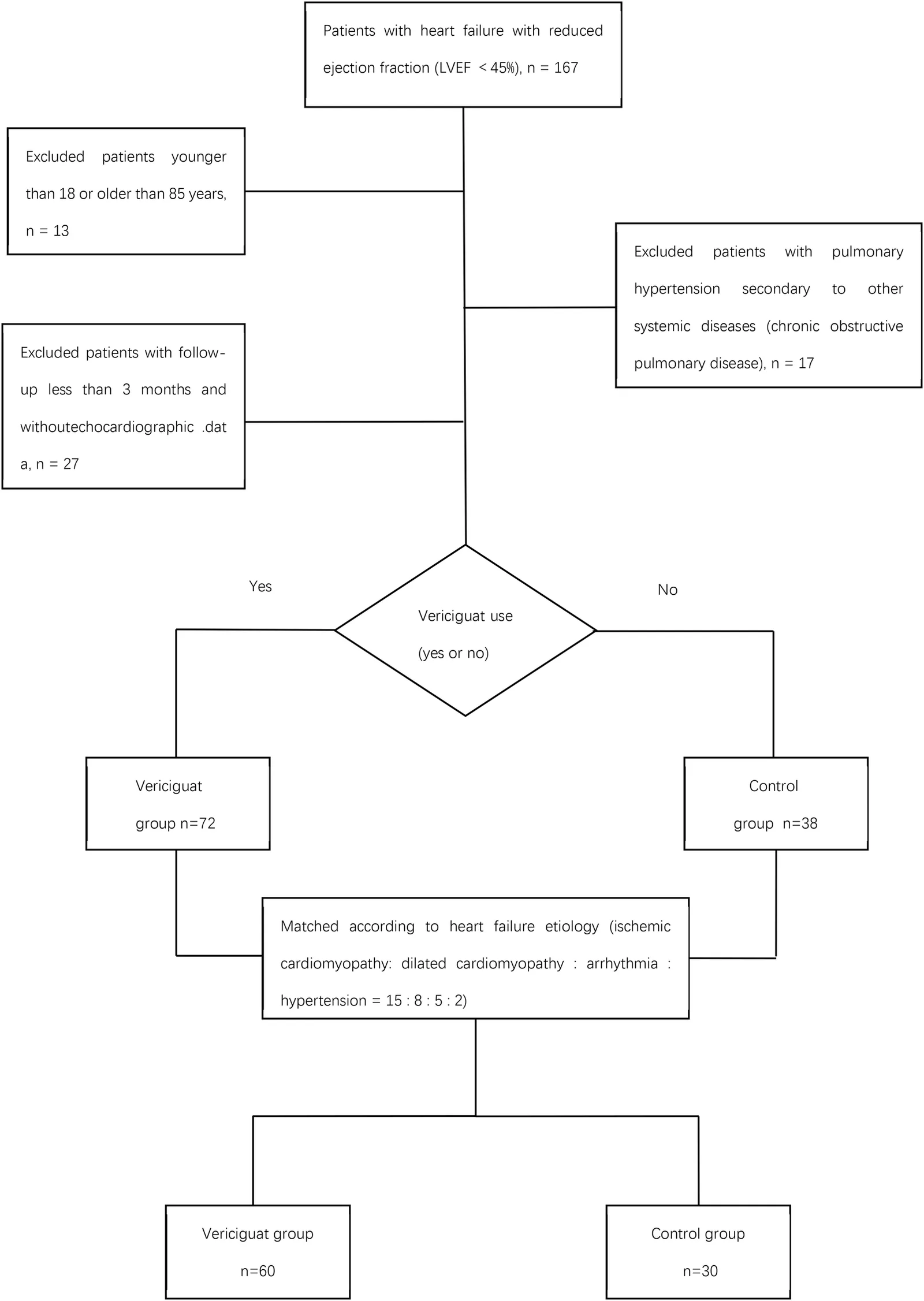
Trial flowchart.

### 2.3 Study endpoints and data collection

Primary Endpoints: Changes in RV function after 3 months of treatment, including tricuspid annular plane systolic excursion (TAPSE), the right ventricular-pulmonary artery coupling index [defined as the ratio of TAPSE to pulmonary artery systolic pressure (PASP), TAPSE/PASP], and changes in PASP.

Secondary Endpoints: Left ventricular (LV) functional improvement, including changes in LVEF, left ventricular end-diastolic dimension (LVEDD), and left ventricular end-systolic dimension (LVESD). Biomarkers: Changes in serum NT-proBNP levels. Clinical events: Rate of heart failure rehospitalization within 3 months.

Safety Endpoints: Incidence of adverse events, including hypotension (systolic blood pressure <90 mmHg or symptomatic), worsening renal function (an increase in serum creatinine >0.5 mg/dL or >50% from baseline), and hyperkalemia (serum potassium >5.5 mmol/L).

Echocardiographic Parameters: Data were retrieved from the hospital's echocardiography database, recording standard echocardiographic parameters for HF patients: left atrial diameter (LAD), LVEDD, LVESD, interventricular septal thickness (IVST), left ventricular posterior wall thickness (LVPWT), LVEF, right atrial transverse (RAT), right atrial longitudinal (RAL), and right ventricular (RV) basal diameter. Cardiac chamber dimensions were indexed to body surface area (BSA). Left ventricular mass (LVM) was calculated as: LVM = 0.8 × 1.04 × [(LVEDD + LVPWT + IVST)^3−LVEDD^3] + 0.6;LVMI was calculated as: LVMI = LVM/BSA;LADI was calculated as: LADI = LAD/BSA; LVEDDI was calculated as: LVEDDI = LVEDD/BSA; LVESDI was calculated as: LVESDI = LVESD/BSA. Right atrial longitudinal dimension index (RALDI) was calculated as: RALDI = RAL/BSA;right atrial transverse dimension index (RATDI) was calculated as: RATDI = RAT/BSA;right ventricular basal diameter index (RVBDI) was calculated as: RVBDI = RV basal diameter/BSA. In the apical four-chamber view, transmitral Doppler was used to measure early (E) and late (A) diastolic peak velocities. Tissue Doppler imaging assessed early (e'), late (a'), and systolic (s') peak velocities at the lateral mitral annulus, from which the E/e' ratio was derived. Continuous-wave Doppler measured peak tricuspid regurgitation velocity (TR). PASP was estimated using the Bernoulli equation applied to TR velocity plus estimated right atrial pressure. RV function indices: TAPSE (normal ≥17 mm; <17 mm indicates RV systolic dysfunction). TAPSE/PASP represents right ventricle-pulmonary artery (RV-PA) coupling, with values < 0.36 indicating poor RV compensatory capacity.

### 2.4 Statistical analysis

Continuous variables with a normal distribution are expressed as mean ± standard deviation (Mean ± SD) and compared between groups using the independent-samples t test. Non-normally distributed data are presented as median (interquartile range) [M (Q1, Q3)] and compared using the Mann–Whitney U test. Within-group comparisons before and after treatment were performed using paired t tests or Wilcoxon signed-rank tests, as appropriate. To address potential baseline imbalance and regression to the mean, between-group differences in echocardiographic outcomes at 3 months were evaluated using separate analysis of covariance models. A multivariable linear regression model was used to examine the association between the change in TAPSE/PASP after 3 months of treatment (ΔTAPSE/PASP) as the dependent variable and changes in left ventricular function parameters (ΔLVEF, ΔLVEDDI), baseline clinical characteristics (age, gender),baseline echocardiographic parameters(LVEF, LVEDDI, TAPSE/PASP). All statistical analyses were conducted using IBM SPSS Statistics version 25. A two-sided *P* value <0.05 was considered statistically significant.

## 3 Results

### 3.1 Baseline clinical characteristics

A total of 90 eligible HF patients with LVEF <45% were included, of whom 60 patients received vericiguat combined with GDMT (vericiguat group) and 30 patients received GDMT alone (control group). The baseline characteristics were summarized in Table 1. There were no significant differences between the vericiguat and control group on the baseline data.

**Table 1:** Baseline clinical characteristics of the vericiguat and control groups.

Characteristic	Overall		*p* value
	Control, *n* = 30	Vericiguat, *n* = 60	
Age, year	73 (53, 79)	69 (55, 77)	0.206
Gender, *n*(%)			0.446
Female	7 (23.3%)	10 (16.7%)	
Male	23 (76.7%)	50 (83.3%)	
Diabetes, *n*(%)	10 (33.3%)	16 (26.7%)	0.511
Hypertension, *n*(%)	21 (70.0%)	31 (51.7%)	0.097
Diuretics, *n*(%)	25 (83.3%)	52 (86.7%)	0.753
MRA, *n*(%)	22 (73.3%)	45 (75.0%)	0.864
RASI, *n*(%)	23 (76.7%)	34 (56.7%)	0.063
SGLT2i, *n*(%)	20 (66.7%)	47 (78.3%)	0.232
BB, *n*(%)	28 (93.3%)	54 (90.0%)	0.714
BSA, m^2^	1.69 (1.55, 1.84)	1.79 (1.66, 1.98)	0.074
SBP, mmHg	128 (121, 150)	131 (118, 145)	0.966
DBP, mmHg	79.87 ± 12.57	81.72 ± 18.95	0.63
Heart rate, bpm	84 (70, 100)	89 (76, 101)	0.475
NT-proBNP, pg/mL	5,157 (1,540, 11,529)	4,245 (1,751, 9,027)	0.558
WBC, 10^9^/L	6.55 (5.8, 8.67)	7.5 (5.8, 10.7)	0.428
HB, g/L	131.6 ± 20.23	137.57 ± 24.38	0.321
PLT, 10^9^/L	195 (138, 234)	191 (146, 236)	0.918
hs-CRP, mg/L	5.3 (2.08, 7.45)	6.3 (4.83, 14.25)	0.116
ALT, u/L	22 (15.5, 32)	24 (14.25, 44)	0.366
AST, u/L	21.5 (16, 31)	26.5 (17, 38.5)	0.169
Urea, mmol/L	7.45 (5.65, 11.35)	7.15 (5.4, 10.48)	0.694
Creatinine, μmol/L	95.5 (72, 135.5)	93.5 (73.25, 135.5)	0.999
eGFR, mL/min/1.73m^2^	68.65 (34.25, 94)	71.5 (47, 94.5)	0.406
Uric acid, μmol/L	416.40 ± 185.43	479.43 ± 160.97	0.100
Potassium, mmol/L	4.02 ± 0.53	3.95 ± 0.50	0.532
LADI, mm/m^2^	28.32 ± 4.57	27.62 ± 5.07	0.523
LVEDDI, mm/m^2^	36.07 ± 4.77	34.96 ± 5.34	0.322
LVESDI, mm/m^2^	29.62 ± 4.95	29.09 ± 5.51	0.656
LVMI, g/m^2^	140.07 ± 28.81	140.60 ± 39.47	0.948
LVEF, %	33.11 ± 7.50	32.13 ± 8.02	0.577
E, m/s	0.85 (0.67, 1.04)	0.86 (0.6, 1.04)	0.678
A, m/s	0.81 ± 0.19	0.72 ± 0.24	0.086
E/A	1.14 (0.77, 1.38)	1.22 (0.78, 1.61)	0.416
s', cm/s	7.00 (6.00, 7.25)	6.00 (5.00, 7.75)	0.349
e', cm/s	7.43 ± 3.20	7.49 ± 3.01	0.933
a', cm/s	8.8 ± 1.63	7.81 ± 2.73	0.071
E/e'	12.54 (8.71, 15.08)	11 (9, 15)	0.543
TAPSE, mm	17.69 ± 2.93	17.95 ± 3.02	0.704
TR, m/s	2.65 ± 0.43	2.80 ± 0.60	0.202
PASP, mmHg	36.52 ± 10.88	41.47 ± 16.25	0.144
TAPSE/PASP	0.53 ± 0.19	0.51 ± 0.22	0.650
RALDI, mm/m^2^	30.55 ± 4.90	30.08 ± 5.80	0.705
RATDI, mm/m^2^	24.78 ± 4.05	23.21 ± 5.13	0.148
RVBDI, mm/m^2^	23.03 ± 3.47	22.48 ± 3.96	0.519

MRA: mineralocorticoid receptor antagonists; RASI, Renin-angiotensin system inhibitors (including ACEI, ARB, and ARNI); SGLT2i, Sodium-glucose cotransporter-2 inhibitors; BB, *β*-blockers; BSA, Body Surface Area; SBP, Systolic Blood Pressure; DBP, Diastolic Blood Pressure; WBC, White Blood Cell; HB, Hemoglobin; PLT, Platelet; hs-CRP, high-sensitivity C-Reactive Protein; ALT, Alanine Transaminase; AST, Aspartate Transaminase; TAPSE, Tricuspid annular plane systolic excursion; TR, Tricuspid Regurgitation Velocity; PASP, Pulmonary Arterial Systolic Pressure; RALDI, Right atrial longitudinal dimension index; RATDI, Right atrial transverse dimension index; RVBDI, right ventricular basal diameter index.

### 3.2 Changes in laboratory parameters during follow-up

All patients completed the 3-month follow-up. Changes in laboratory parameters are shown in Table 2. In the vericiguat group, after 3 months of treatment, systolic blood pressure, diastolic blood pressure, serum NT-proBNP, high-sensitivity C-reactive protein (hs-CRP), white blood cell count, hemoglobin, ALT, AST, serum creatinine, and uric acid levels were all significantly reduced compared with baseline. In contrast, estimated glomerular filtration rate (eGFR) and serum potassium levels increased (*p* < 0.05); however, potassium levels remained within the normal reference range (3.5–5.5 mmol/L), and no cases of hyperkalemia were reported during the observation period. In the control group, after 3 months of standard GDMT therapy, systolic blood pressure, diastolic blood pressure, serum NT-proBNP, ALT decreased, while serum potassium levels increased (*p* < 0.05), remaining within the normal range.

**Table 2:** Changes in clinical and laboratory parameters at 3 months in the vericiguat and control groups.

Parameter	Control		*p*	Vericiguat		*p*
	Pre	Post		Pre	Post	
SBP, mmHg	128 (121, 150)	121 (113, 135)	0.001	131 (118, 145)	121 (101, 131)	<0.001
DBP, mmHg	79.87 ± 12.57	73.60 ± 8.92	0.006	81.72 ± 18.95	74.70 ± 12.83	<0.001
NT-proBNP, pg/mL	5,157 (1,540, 11,529)	1,380 (537, 4,532)	<0.001	4,245 (1,751, 9,027)	1,195 (657, 3,127)	<0.001
WBC, 10^9^/L	6.55 (5.8, 8.67)	7.3 (5.75, 8.625)	0.946	7.5 (5.8, 10.7)	7.0 (5.8, 8.1)	0.014
HB, g/L	131.6 ± 20.23	126.43 ± 23.19	0.116	137.57 ± 24.38	132.47 ± 21.65	0.008
PLT, 10^9^/L	195 (138, 234)	172 (145, 262)	0.641	191 (146, 236)	178 (144, 230)	0.439
hs-CRP, mg/L	5.3 (2.08, 7.45)	5.1 (1.88, 6.1)	0.226	6.3 (4.83, 14.25)	5.3 (2.6, 10)	0.001
ALT, u/L	22 (15.5, 32)	17 (11, 30)	0.025	24 (14.25, 44)	20 (14, 28.75)	0.016
AST, u/L	21.5 (16, 31)	18 (15.75, 31.75)	0.516	26.5 (17, 38.5)	20 (17, 24.75)	0.001
Urea, mmol/L	7.45 (5.65, 11.35)	8.8 (6.43, 11.95)	0.012	7.15 (5.4, 10.48)	7.2 (5.6, 8.98)	0.526
Creatinine, μmol/L	95.5 (72, 135.5)	92.5 (73.5, 163)	0.446	93.5 (73.25, 135.5)	83.5 (67, 122)	0.020
eGFR, mL/min/1.73m^2^	68.65 (34.25, 94)	65.5 (36.5, 94.75)	0.398	71.5 (47, 94.5)	79.5 (48.3, 96)	0.044
Uric acid, μmol/L	416.4 ± 185.43	390.87 ± 129.07	0.331	479.43 ± 160.97	383.83 ± 154.84	<0.001
Potassium, mmol/L	4.02 ± 0.53	4.29 ± 0.62	0.045	3.95 ± 0.50	4.11 ± 0.40	0.026

### 3.3 Echocardiographic findings at 3-month follow-up

Left heart structure and function: Improvements in left ventricular remodeling were observed in both groups. In both the vericiguat and control groups, LVEDDI, LVESDI and LVEF showed significant improvements from baseline to 3 months (within-group *p* < 0.05).

Right heart function and pulmonary artery pressure: In the vericiguat group, right ventricular function improved significantly after treatment. The TAPSE/PASP ratio [0.51 ± 0.22] increased significantly at 3 months [0.60 ± 0.22, *p* = 0.001]. Right heart structure also improved: RALDI significantly decreased [baseline 30.07 ± 5.80 mm/m2 vs. 28.57 ± 5.43 mm/m2 at follow-up, *p* = 0.001], as did the RATDI [23.21 ± 5.13 mm/m^2^ vs. 21.86 ± 4.40 mm/m^2^, *p* = 0.012] and right ventricular basal diameter index [22.48 ± 3.96 mm/m2 vs. 21.07 ± 3.59 mm/m2, *p* < 0.001]. PASP decreased significantly [41.47 ± 16.25 mmHg vs. 36.20 ± 14.32 mmHg, *p* = 0.006]. In contrast, in the control group, no significant changes were observed in right heart function parameters (TAPSE, TAPSE/PASP, PASP) or chamber dimensions (RALDI, RATDI, RVBDI) at 3 months compared with baseline (within-group *p* > 0.05),as shown in Table 3.

**Table 3:** Changes in echocardiographic parameters at 3 months between the vericiguat and control groups.

	Control			Vericiguat		
Parameter	Pre	Post	*p*	Pre	Post	*p*
LADI, mm/m^2^	28.32 ± 4.57	27.76 ± 4.68	0.371	27.62 ± 5.07	26.30 ± 4.83	0.001
LVEDDI, mm/m^2^	36.07 ± 4.77	34.60 ± 5.70	0.011	34.96 ± 5.34	33.98 ± 5.87	0.018
LVESDI, mm/m^2^	29.62 ± 4.95	27.42 ± 5.96	0.006	29.09 ± 5.51	26.74 ± 6.70	＜0.001
LVEF, /%	33.11 ± 7.50	40.73 ± 11.99	＜0.001	32.13 ± 8.02	40.76 ± 12.34	＜0.001
LVMI, g/m^2^	140.07 ± 28.81	133.57 ± 33.92	0.147	140.60 ± 39.47	130.24 ± 34.82	0.015
E, m/s	0.89 ± 0.33	0.78 ± 0.34	0.083	0.86 (0.6, 1.04)	0.67 (0.51, 0.90)	0.003
A, m/s	0.81 ± 0.19	0.83 ± 0.0.20	0.586	0.72 ± 0.24	0.80 ± 0.25	0.041
E/A	1.16 ± 0.52	1.11 ± 0.52	0.198	1.22 (0.78, 1.61)	0.82 (0.57, 1.39)	0.004
s', cm/s	7 (6, 7.25)	7 (6, 9)	0.268	6 (5, 7.75)	7 (6, 8.83)	0.003
e', cm/s	7.43 ± 3.20	7.38 ± 2.30	0.928	7.49 ± 3.01	7.33 ± 2.82	0.749
a', cm/s	8.8 ± 1.62	9.12 ± 2.46	0.429	7.81 ± 2.73	8.82 ± 2.46	0.028
E/e'	12.54 (8.71, 15.08)	10.31 (7.44, 15.45)	0.136	12.27 ± 5.45	10.97 ± 5.17	0.119
TAPSE, mm	17.69 ± 2.93	18.28 ± 2.95	0.412	17.95 ± 3.02	19.00 ± 2.72	0.023
TR, m/s	2.65 ± 0.43	2.83 ± 0.50	0.072	2.8 ± 0.59	2.66 ± 0.53	0.050
PASP, mmHg	36.52 ± 10.88	41.11 ± 15.10	0.12	41.47 ± 16.25	36.20 ± 14.32	0.006
TAPSE/PASP	0.53 ± 0.19	0.50 ± 0.18	0.414	0.51 ± 0.22	0.60 ± 0.22	0.001
RALDI, mm/m^2^	30.55 ± 4.90	31.14 ± 4.52	0.476	30.07 ± 5.80	28.57 ± 5.43	0.001
RATDI, mm/m^2^	24.78 ± 4.05	24.57 ± 3.78	0.783	23.21 ± 5.13	21.86 ± 4.40	0.012
RVBDI, mm/m^2^	23.03 ± 3.47	23.21 ± 3.63	0.755	22.48 ± 3.96	21.07 ± 3.59	＜0.001

To account for potential baseline imbalance and regression to the mean, the 3-month echocardiographic outcomes were compared using baseline-adjusted ANCOVA. The adjusted TAPSE/PASP ratio was significantly higher in the vericiguat group than in the control group [0.608 (95%CI 0.561–0.654) vs. 0.494 (95%CI 0.428–0.560); adjusted mean difference=0.113, 95%CI 0.033–0.194; F = 7.817, *P* = 0.006]. Adjusted PASP was significantly lower in the vericiguat group [35.392 (95%CI 32.125–38.659) vs. 42.716 (95%CI 38.077–47.355) mmHg; adjusted mean difference=−7.324 mmHg, 95%CI −13.029 to −1.620; F = 6.512, *P* = 0.012], whereas no significant between-group difference was observed in TAPSE (*P* = 0.276). The vericiguat group also had significantly lower RALDI (adjusted mean difference=−2.241 mm/m^2^, 95%CI −3.746 to −0.737; *P* = 0.004), RATDI (−1.897 mm/m^2^, 95%CI −3.425 to −0.369; *P* = 0.016), and RVBDI (−1.767 mm/m^2^, 95%CI −2.878 to −0.655; *P* = 0.002). In contrast, no significant adjusted between-group differences were observed in LVEDDI, LVESDI, or LVEF (all *P* > 0.05), as shown in Table 4.

**Table 4:** Baseline-adjusted between-group comparisons of echocardiographic outcomes at 3 months.

Parameter	Adjusted control mean (95%CI)	Adjusted vericiguat mean (95%CI)	Adjusted mean difference (95%CI)	F	*P* value
TAPSE/PASP	0.494 (0.428, 0.560)	0.608 (0.561, 0.654)	0.113 (0.033, 0.194)	7.817	0.006
TAPSE, mm	18.312 (17.315, 19,309)	18.986 (18.281, 19,690)	0.673(−0.548, 1.895)	1.201	0.276
PASP, mmHg	42.716 (38.077, 47.355)	35.392 (32.125, 38.659)	−7.324(−13.029, −1.620)	6.512	0.012
RALDI, mm/m^2^	30.919 (29.691, 32.147)	28.677 (27.809, 29.545)	−2.241(−3.746, −0.737)	8.769	0.004
RATDI, mm/m^2^	24.026 (22.784, 25.269)	22.129 (21.254, 23.004)	−1.897(−3.425, −0.369)	6.092	0.016
RVBDI, mm/m^2^	22.964 (22.057, 23.871)	21.197 (20.557, 21.838)	−1.767(−2.878, −0.655)	9.975	0.002
LVEDDI, mm/m^2^	33.890 (32.773, 35.007)	34.333 (33.544, 35.121)	0.442(−0.928, 1.813)	0.412	0.523
LVESDI, mm/m^2^	27.074 (25.628, 28.520)	26.912 (25.891, 27.934)	−0.162(−1.933, 1.609)	0.033	0.856
LVEF, %	40.173 (36.422, 43.923)	41.035 (38.385, 43.686)	0.863(−3.733, 5.459)	0.139	0.71

Data are presented as estimated marginal means with 95% confidence intervals (CIs). Separate analysis of covariance (ANCOVA) models were performed for each outcome, with the 3-month value as the dependent variable, treatment group as the fixed factor, and the corresponding baseline value as the covariate. Adjusted control mean and adjusted vericiguat mean refer to the estimated 3-month marginal means after adjustment for the corresponding baseline measurement. Adjusted mean difference was calculated as the vericiguat group minus the control group. Therefore, a positive value indicates a higher adjusted value in the vericiguat group, whereas a negative value indicates a lower adjusted value in the vericiguat group. F and *P* values represent the baseline-adjusted between-group effect of treatment.

### 3.4 Factors associated with right ventricular improvement

In an exploratory multivariable linear regression analysis restricted to the vericiguat group, baseline TAPSE/PASP was independently and inversely associated with change in TAPSE/PASP (B = −0.485, 95%CI −0.719 to −0.252, *P* < 0.001), indicating greater improvement among patients with lower baseline RV–PA coupling. Sex was significantly associated with ΔTAPSE/PASP (B = 0.136, 95%CI 0.004–0.268, *P* = 0.043), with female patients showing a greater increase in TAPSE/PASP than male patients after adjustment for the other covariates. Change in LVEF showed a nonsignificant positive association (B = 0.005, standardized β=0.258, *P* = 0.075), whereas age, baseline LVEF, baseline LVEDDI, and change in LVEDDI were not significantly associated with change in TAPSE/PASP, as shown in Table 5.

**Table 5:** Multivariable linear regression analysis of ΔTAPSE/PASP in vericiguat group.

	Multivariable analysis	
Covariate	*β* (95%CI)	*p* value
Gender	0.136 (0.004, 0.268)	0.043
Age	−0.001 (−0.005, 0.003)	0.665
Baseline LVEF	−0.003 (−0.012, 0.007)	0.587
ΔLVEF	0.005 (−0.001, 0.011)	0.075
Baseline LVEDDI	−0.004 (−0.020, 0.013)	0.647
ΔLVEDDI	0.006 (−0.013, 0.025)	0.541
Baseline TAPSE/PASP	−0.485 (−0.719, −0.252)	<0.001

### 3.5 Clinical outcomes

By the end of follow-up, the mean vericiguat dose in the treatment group was 8.67 mg/day, and 75% of patients had reached the target dose of 10 mg/day. During the 3-month follow-up, heart failure rehospitalization occurred in 17 of 60 patients (28.3%) in the vericiguat group and 14 of 30 patients (46.7%) in the control group. The rehospitalization rate was numerically lower in the vericiguat group, but the between-group difference did not reach statistical significance (RR = 0.61, 95%CI = 0.35–1.06; *χ*^2^ = 2.98, *P* = 0.084), as shown in Figure 2. During the 3-month follow-up, none of the prespecified safety endpoints occurred. No patient developed hypotension, worsening renal function, or hyperkalemia. No new safety signals were identified during the study period.

**Figure 2:**
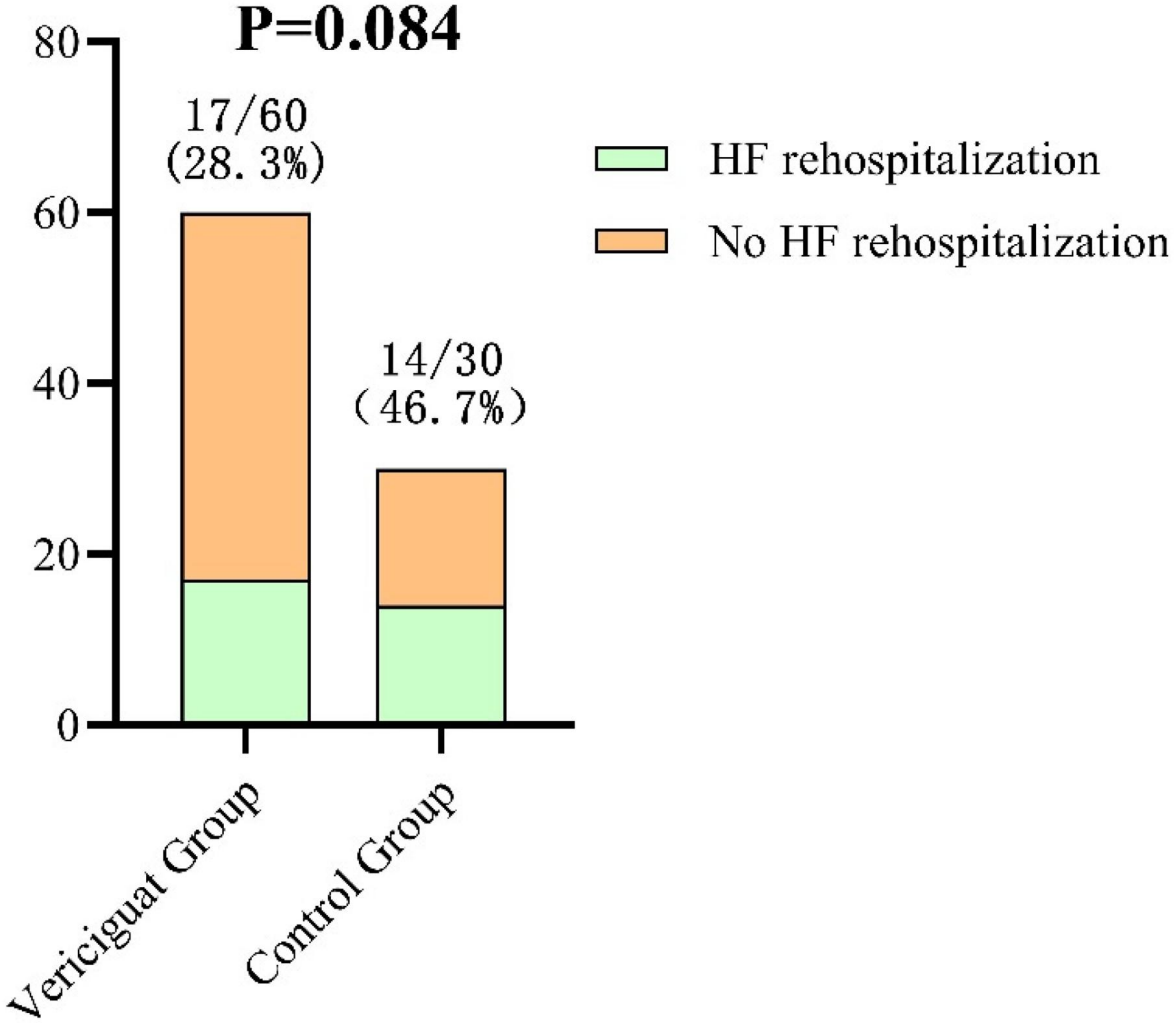
Heart failure rehospitalization within 3 months in the control and vericiguat groups.

A retrospective power analysis showed that the current sample provided approximately 40% power to detect the observed difference at a two-sided *α* level of 0.05. Assuming the same event rates and a 2:1 allocation ratio, approximately 243 patients would be required to achieve 80% power. Therefore, the rehospitalization analysis should be considered exploratory, and the nonsignificant result may reflect limited statistical power rather than the absence of a clinically meaningful difference.

## 4 Discussion

This study was a single-center retrospective observational investigation evaluating the effects of vericiguat on right ventricular function in patients with HFrEF. The main findings were as follows: (1) Addition of vericiguat for 3 months on top of standard quadruple GDMT significantly improved right heart structure and function in HFrEF patients, evidenced by a marked reduction in PASP, a significant increase in the right ventricle-pulmonary artery coupling index (TAPSE/PASP), and notable decreases in right atrial and right ventricular diameters;(2) In the vericiguat group, neither baseline LVEF and LVEDDI nor their changes during follow-up were significantly associated with ΔTAPSE/PASP in the multiple linear regression analysis. These findings suggest that the increase in ΔTAPSE/PASP was not significantly associated with changes in left ventricular function. (3) The short-term (3-month) clinical benefit of vericiguat was mainly reflected by improvements in right heart structure and function, without a significant reduction in heart failure rehospitalization compared to GDMT alone; (4) Vericiguat combined therapy was well tolerated with no newly observed safety concerns.

Vericiguat, a soluble guanylate cyclase (sGC) stimulator, exerts its core mechanism by enhancing the sGC-cGMP pathway, thereby improving endothelial function, vascular relaxation, and myocardial fibrosis (6). Previous studies, including the VICTORIA trial, have demonstrated its beneficial effects on left ventricular systolic function and overall prognosis in HFrEF patients, particularly regarding the composite endpoint of cardiovascular death and heart failure hospitalization (7, 10). A study also showed that adding vericiguat for 6 months to conventional GDMT (not necessarily the full quadruple regimen) resulted in significant left ventricular reverse remodeling, reflected by decreases in LVEDD and LVESD and improved LVEF (11). Our study similarly observed reductions in left ventricular size and increases in LVEF in both groups after 3 months of GDMT, regardless of vericiguat use. However, the vericiguat group did not exhibit superior improvement in these left heart parameters. This partially aligns with Fujii's findings that patients not receiving optimized quadruple therapy experienced more pronounced left ventricular reverse remodeling with vericiguat, suggesting that when GDMT is already optimized,the short-term additional benefit of vericiguat on left heart function may be limited or require a longer duration to manifest (12).

The most important finding of this study lies in the potential benefit of vericiguat on right heart function. Research exploring the direct effects of vericiguat on the right heart has been very limited to date. A recent single-center Japanese study reported that vericiguat improved adverse left ventricular remodeling and RV-PA uncoupling in HFrEF patients (13), but lacked a control group for comparison. Our study, by including a rigorously matched control group receiving standard quadruple GDMT concurrently, more clearly delineates the specific role of vericiguat in improving right heart structure and function: despite similar degrees of left heart improvement, patients treated with vericiguat showed significant reductions in right atrial and right ventricular diameters, marked decreases in PASP, and significant increases in TAPSE/PASP, changes not observed in the control group. Multiple linear regression further demonstrated that the improvement in RV-PA coupling (ΔTAPSE/PASP) was not significantly associated with left heart functional changes (ΔLVEF, ΔLVEDDI), suggesting that the right heart benefits of vericiguat may be independent of left heart improvements and potentially mediated via direct effects on the right heart, pulmonary vasculature, or endothelial function.

The underlying mechanisms warrant further investigation. Activation of the sGC-cGMP pathway exerts pleiotropic effects (14–16): (1) Pulmonary vasodilation: By stimulating cGMP production, it directly promotes relaxation of pulmonary vascular smooth muscle, thereby reducing pulmonary vascular resistance and right ventricular afterload (PASP), which likely represents a major mechanism for the observed PASP reduction; (2) Anti-right ventricular remodeling and fibrosis: cGMP signaling is also present in cardiomyocytes, where activation of this pathway may inhibit myocardial hypertrophy and fibrosis-related signaling cascades such as TGF-β and NFAT, attenuating maladaptive right ventricular remodeling under pressure overload, potentially explaining the reduction in right atrial and ventricular size; (3) Improvement of right ventricular myocardial metabolism and contractile function: cGMP modulates energy metabolism and calcium homeostasis, potentially enhancing right ventricular contractility (reflected by stable or improved TAPSE) and efficiency, ultimately improving RV-PA coupling (increased TAPSE/PASP) (13); (4) Systemic anti-inflammatory and antifibrotic effects: The systemic inflammatory state in HFrEF adversely affects multiple organs (17); vericiguat may confer protective effects on the right heart and pulmonary vasculature by mitigating systemic inflammation-induced damage.

It is noteworthy that although activation of the NO signaling pathway is generally beneficial for pulmonary hypertension (PH) and right heart function, some studies have suggested that inhaled NO may increase left ventricular filling pressures (PAWP) in patients with left ventricular dysfunction (18), and elevated PAWP is associated with adverse outcomes (19). Our study demonstrated that vericiguat significantly reduced PASP. This finding is supported by a recent small invasive study by Suzuki et al (20), who performed right heart catheterization in 12 HFrEF patients treated with vericiguat and observed reductions in both mean pulmonary artery pressure (mPAP) and PAWP on days 2–3 after treatment initiation. These results are consistent with vericiguat has favorable hemodynamic tolerability without inducing potentially harmful increases in PAWP.

Regarding clinical benefits, the VICTORIA trial established that vericiguat significantly reduced the risk of the primary composite endpoint of cardiovascular death or heart failure hospitalization over a median follow-up of 10.8 months (7). In our cohort of standard HFrEF patients, one key endpoint was heart failure rehospitalization within 3 months. We observed a numerically lower rehospitalization rate in the vericiguat group (28.3%) compared to the control group (46.7%), although this difference did not reach statistical significance (*p* = 0.084). These findings suggest that although vericiguat added to optimized GDMT can achieve early improvements in right heart structure and function, these changes did not translate into significant early clinical symptom relief or reduced rehospitalization within 3 months. This may be due to the relatively short follow-up duration, during which effects on hard clinical endpoints may not yet be fully apparent, or it may imply that early right heart functional improvements require longer to manifest as clinical benefits. Extended follow-up periods will be necessary to clarify the potential impact of vericiguat on rehospitalization rates and survival.

## 5 Conclusion

This study demonstrates that adding vericiguat to optimized standard quadruple GDMT for 3 months significantly reduces pulmonary artery systolic pressure (PASP), improves right ventricle-pulmonary artery coupling (TAPSE/PASP), and promotes structural reverse remodeling of the right atrium and right ventricle (reduced chamber diameters) in patients with HFrEF. These right heart benefits occur independently of changes in left ventricular structure and function. Vericiguat showed good short-term safety. However, early improvements in right heart structure and function over 3 months did not result in significant reductions in heart failure rehospitalization compared to GDMT alone. The long-term clinical benefits of vericiguat on right heart function require confirmation in larger, prospective studies with longer follow-up.

This study has several limitations. First, it is a single-center, retrospective, observational study rather than a randomized controlled trial (RCT), which may introduce selection bias and confounding factors. Second, the study was not adequately powered to evaluate heart failure rehospitalization. Although the observed rehospitalization rate was numerically lower in the vericiguat group, the wide confidence interval included both a potentially meaningful benefit and no difference. This clinical outcome should therefore be regarded as exploratory and requires confirmation in a substantially larger cohort. Third, right heart catheterization was not performed; therefore, direct hemodynamic parameters such as mPAP, PAWP, PVR, and cardiac output were unavailable, precluding precise assessment of vericiguat's effects on pulmonary vascular resistance and overall right heart hemodynamics. Fourth, detailed information on vericiguat dosing and titration was lacking, preventing analysis of dose-response relationships. Fifth, Comprehensive assessment of right ventricular morphology and systolic function, including RV fractional area change, tricuspid annular s' velocity, RV free-wall strain, free-wall thickness, and three-dimensional RV volumes, was not available. Therefore, the present findings mainly reflect changes in estimated pulmonary pressure, RV-PA coupling, and right-heart chamber dimensions rather than a complete assessment of intrinsic RV contractile function. Sixth, Echocardiographic measurements were obtained from routine clinical reports without formal reader blinding or centralized reassessment, and interobserver and intraobserver reproducibility data were unavailable. Therefore, measurement variability and observer-related bias cannot be excluded. Despite these limitations, this study provides preliminary evidence of the potential benefit of vericiguat on right heart function in HFrEF patients and offers valuable data to inform the design of future prospective RCTs focusing on right heart outcomes.

## Data Availability

The original contributions presented in the study are included in the article/Supplementary Material, further inquiries can be directed to the corresponding authors.
